# Molecular approach for identification of *Catla catla* using mitochondrial *CO1* from Pakistan

**DOI:** 10.1080/23802359.2020.1768913

**Published:** 2020-07-25

**Authors:** Zara Naeem, Samrah Masud, Shoaib Hassan, Muhammad Naeem

**Affiliations:** Institute of Pure and Applied Biology, Bahauddin Zakariya University, Multan, Pakistan

**Keywords:** *Catla catla*, COI, DNA barcoding, identification

## Abstract

DNA barcoding is a rapid, precise, and effective way of species identification. A short and standard target gene marker is used to create sequence profile of identified species. Specific tag or marker is used, which is derived from mitochondrial COI for identification. Effectiveness of this method axes the degree of divergence among species. Identification is necessary for their representation. In the present work, *Catla catla* was used to study by using Cytochrome C Oxidase 1.The genetic distances were computed, and Neighbor Joining tree was constructed based on the Kimura 2 Parameter method. GenBank and BOLD revealed definitive identity matches. Conspecific and congeneric K2P nucleotide divergence was estimated. Evolutionary tree was analyzed clearly by relating their species to phylogenetic tree, as same as species were bunched under same tree node, while species were differently clustered under distinct nodes. These findings conclude that the gene sequence may serve as a milestone for identification and phylogenetic history of related species at molecular level.

## Introduction

The identification of fish species is one of the major tasks of taxonomy. The identification of fish species is commonly based on the visible morphology and is carried out using different morphological keys (Ward 2009). DNA barcoding provides speed accuracy in species identification with a focus on analysis on small fragment mitochondrial DNA (Muchlisin et al. 2012). Taxonomic issues can also be solved by these molecular studies. DNA barcoding is known to be a source of species identification (Li et al. 2018). Sequencing is the most important for the species of living creatures since it is a great tool used for this variety and also provides comprehensive information (Singh et al. 2010). Identification of the species based on certain sequences of species of mitochondrial DNA helps the precise identification of unknown species (Dawnay et al. 2007). Main barcode goal is assessed by using Cytochrome C oxidase 1 gene to identified species unknown into known species (Kerr et al. 2009). DNA barcoding becomes known as a molecular method for the identification of species. DNA barcoding relies on specific region of the mitochondrial gene being sequenced, amplified, and analyzed comparison. Molecular basis for biological barcode, to identify organism is the central goal of DNA barcoding, used to create a standardized library for DNA based identification of target species (Kerr et al. 2007).

DNA barcoding can correct the field of misidentification, reduces ambiguity for identification of species, exact species identification, and expand taxonomists expertise (Stoeckle et al. 2004). The precise organism identification has been the realm of taxonomic experts and identifies an organism; DNA based identification system uses standardized molecular techniques of DNA extraction, Polymerase chain reaction, and DNA sequencing that is used for identification of an unknown organism (Seifert et al. 2007).

The aim of the present study was to carry out molecular-based identification of *Catla catla* using mitochondrial CO1 from Pakistan.

## Materials and methods

### Fish sampling

A total of 15 samples of *C. catla* were collected from River Chenab (Lat: 30°04′31.33″N, Long: 71°11′31.67″E), Punjab, Pakistan. Samples were identified with the help of standard taxonomic key (Mirza and Sandhu 2007) on the basis of morphological characters. *Catla catla* or *Labeo catla* is Synonyms of *Gibelion catla* as confirmed by various studies, such as Hamilton (1822), Jhingran (1966), Bhuiyan (1964), Shaw and Shebbeare (1937), Rahman (1974), and Menon (1974), and locally used in Pakistan. DNA extraction was carried out by a modified phenol-chloroform method and stored at –20 °C until further analysis. Quantification of extracted DNA was carried out with nanophotometer (IMPLEN) at A_260_/A_280_ nm absorbance. Polymerase chain reaction of extracted DNA for amplification of the identification region was carried out. Polymerase chain reaction was carried out with reaction setting (one cycle of denaturation at 95 °C with respective 40 cycles of denaturation phase at 94 °C, annealing at 55 °C and extension at 72 °C with one cycle of final extension for 7 min. Sequence of primer used for polymerase chain reaction amplification and sequencing by Cytochrome C oxidase 1 gene is given in Table 1. PCR products were powered by 1.5% gels and displayed on BioRed Gel Doc to observe the quality of the product. All the samples were used to extract DNA, but for further analysis only clear extracted DNA was used for sequence purpose. The obtained sequence was analyzed by BOLD system and blast on NCBI to identify the unknown sequence to the known product was sequenced and analyzed by using BioEdit lign (version 7.0.5.3) following the method of Hall (1999). Pairwise genetic distance was used to calculate Kimura 2 Parameter distance (Hebert et al. 2003). Neighbour-Joining (NJ) tree (Saitou and Nei 1987) was constructed with MEGA 5 software (Tamura et al. 2011). Sequence accession number as following as provided by GenBank (MT373809).

**Table 1.** Primers sequences used for PCR amplification and sequencing through CO1 identification gene.

Sr. no.	Primer	Sequence (5′→3′)	TM (°C)	GC%	Primer size (nt)
1	Forward primer	TCAACCAACCACAAAGACATTGGAAC	64.7	46.15	26
2	Reverse primer	TAGACTTCTGGGTGGCCAAAGAATCA	66.3	46.15	26

TM: Melting temperature; nt: Nucleotide (Naeem and Hassan 2019).

## Results

**Figure 1.**
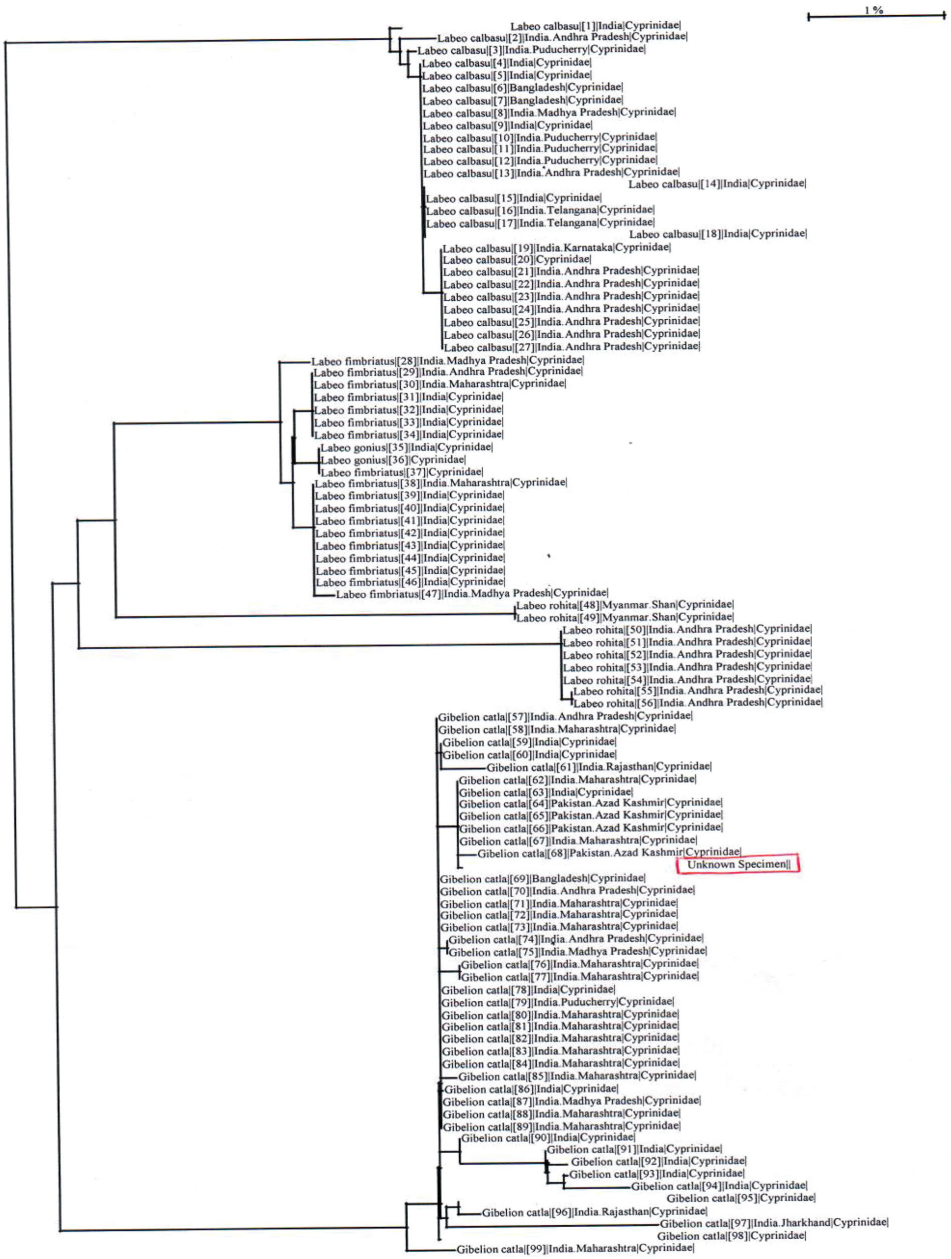
Hierarchical placement of unknown specimen.

Results of the present study revealed that *C. catla* identification with a total read length of consensus sequence was found 641 base pair. Molecular identification of *C. catla* was carried out, and the total read length of consensus was found 641 base pair. Barcode of life data system match found *C. catla* fish species with 100% similarity index. Results for the study used to calculate the genetic distance by Kimura 2 Parameter analysis inter and intraspecific fish species are shown in Table 2. The NJ tree among species is shown in Figure 1, which was marked for distance analysis from BOLD system.

**Table 2.** Kimura 2 parameter distances.

Genetic distance (Kimura 2 parameter percent)					
	Comparison within species (intraspecifice)		Comparison between species (interspecific)		
Level of taxonomy	Genera value (%)	Specie value (%)	Distance variance (%)	Maximum (%)	Average value (%)
Species values	6.72	0.38	0.04	0.65	0.43

## Discussion

DNA barcoding allows for identification of species and also shared organisms significant interspecies differences DNA barcoding breaks through the over-reliance on the experiences of taxonomists in traditional morphological classification of species identification. The mitochondrial COI gene exhibits high levels of conservation, genetic variability between different species, is usually utilized as a species barcode, and its high efficiency in species identification as marked in the present study.

A short 648 base-pair region in the mtDNA Cytochrome c oxidase 1 gene and its resulting polypeptide CO1 qualifies to serve as a standardized DNA barcode for all animals (Consortium for the Barcode of Life [CBOL] 2009) in accordance with the above characteristics. The gene has successfully served as a barcode in many different animals such as birds, fish, and insects (Hebert et al. 2004; Ward et al. 2005; Hajibabaei et al. 2006), mainly because it is well conserved, showing low levels of variance, within a species but it is at the same time showing enough divergence between species to allow for differentiation among many different species (Hebert et al. 2003).

Comparing results obtained from database NCBI and BOLD revealed that the identification of species was different. Results were also supported by percentages on similarity basis and phylogenetic tree analysis and limited to correct identification of the species (Ward et al. 2005). 641 base pair sequence of *C. catla* was used for identification region in the present study as 650 base pair CO1 gene sequence was studied by Lohman et al. (2009), and a 649 base pair used to identify the species by Naeem and Hassan (2019). The primary purpose of molecular study is to identify unknown species (Kerr et al. 2009).

Hybridization may also problematic for DNA barcoding species identifications (Hebert et al. 2003, 2004; Mitchell 2008; Ward 2009). Occasionally, a complex relationship between the same species results in failures for the identification of species using DNA barcoding. Marshall et al. (2008) found that there was no species gap within the mitochondrial data to establish a threshold between intra and interspecific variation. The findings of this study add support to previous studies’ conclusions that the barcoding gap does not always as phenomenal (Lukhtanov et al. 2009; Ward 2009). The efficiency of species identification through DNA barcoding depends on both interspecific divergence and intraspecific divergence (Bhattacharjee et al. 2012). In this study, the average intraspecific K2P distance was 0.38%, compared with 6.72% for species within genera as it is many-fold higher than the mean intraspecific distance as justified by with the number of freshwater fish in Indonesia (0.15% and 2.53%, respectively) (Muchlisin et al. 2012), Canadian freshwater fish (0.27% and8.37%, respectively) (Hubert et al. 2008), and Australian marine fish (0.39% and 9.93%, respectively) (Ward et al. 2005); this result corresponds to the DNA barcoding principle that interspecific divergence sufficiently outscores intraspecific divergence. Comparison (260/280 nm) of 1.6–2 for clear DNA extraction product (Cawthorn et al. 2011) showed that the quality of DNA was found 1.6–1.767. These results of the current study were found in general agreement with the results of Naeem and Hassan (2019). In this study, the average K2P of intraspecific species was 0.2%, compared to 6.50% for genera. Kenchington et al. (2017) concluded with the principle of DNA indicating that the interspecific separation is sufficient to exchange the differences. In addition, the difference was greater than the 13.9 differences that were mentioned by the marine fish that often met the Atlantic of Canada. In total, the total number of 639 base pairs were found, the size and the average K2P found to be 0, 1.41, and 0.2%, respectively, within their types, as so 0.11, 1.82, and 0.34% (Naeem and Hassan 2019) available to *C. catla* found in general agreement.

The results of this study reveal that the DNA barcoding has succeeded in identifying the fish species. The DNA-based description of the genre can be used to assess the fish variety and to assess the production of fish (Takahara et al. 2013). This approach will provide guidance for future fish stocks that are required to be deposited. Once the DNA barcode database is established, the scientific and practical benefits of fishing will be different. As DNA barcoding can distinguish all types of fish and distinguish between the eggs, the larva, and the species, hence, the results will provide more information about the different types of fish to sailors and environmental experts who are responsible for maintenance and sustainable use of fish resources.

## Conclusion

DNA barcoding of a functional fish species identification technique compares the different conventional methods. Previously used identification techniques have several limitations that do not work in developmental body stages of fish, processed, fillet, and in case of specimen damaged. DNA barcoding is based on *CO1* gene of mitochondrial DNA and has enough variability to differentiate the species. Also, it estimates the nucleic divergence among species, genus, and family. Moreover, DNA barcoding in the field of taxonomy is a useful tool for fish identification.

## Data Availability

The data that support the finding of this study is openly available in the National Center for Biotechnology Information (NCBI) at https://www.ncbi.nlm.nih.gov/nuccore/MT373809, reference number (MT373809).
